# An antibiotic’s journey from marketing authorization to use, Norway

**DOI:** 10.2471/BLT.16.172874

**Published:** 2017-01-19

**Authors:** Christine Årdal, Hege Salvesen Blix, Jens Plahte, John-Arne Røttingen

**Affiliations:** a Norwegian Institute of Public Health, Postboks 4404, Nydalen, Oslo 0403, Norway.

## Abstract

Here we describe in detail marketing authorization and reimbursement procedures for medicinal products in Norway, with particular reference to nine novel antibiotics that received marketing authorization between 2005 and 2015. The description illustrates that, in places like Norway, with effective antibiotic stewardship policies and an associated low prevalence of antibiotic-resistant bacterial infection, there is little need for newer, more expensive antibiotics whose therapeutic superiority to existing compounds has not been demonstrated. Since resistance begins to emerge as soon as an antibiotic is used, Norway’s practice of leaving newer antibiotics on the shelf is consistent with the goal of prolonging the effectiveness of newer antibiotics. An unintended consequence is that the country has signalled to the private sector that there is little commercial value in novel antibiotics, which may nevertheless still be needed to treat rare or emerging infections. Every country aims to improve infection control and to promote responsible antibiotic use. However, as progress is made, antibiotic-resistant bacteria should become less common and, consequently, the need for, and the commercial value of, novel antibiotics will probably be reduced. Nevertheless, antibiotic innovation continues to be essential. This dilemma will have to be resolved through the introduction of alternative reward systems for antibiotic innovation. The DRIVE-AB (Driving re-investment in research and development and responsible antibiotic use) research consortium in Europe has been tasked with identifying ways of meeting this challenge.

## Résumé

Nous décrivons ici en détail les procédures d'autorisation de mise sur le marché et de remboursement des médicaments en Norvège, en particulier de neuf nouveaux antibiotiques qui ont reçu une autorisation de mise sur le marché entre 2005 et 2015. Cette description montre que, dans les pays comme la Norvège qui ont adopté des politiques d'utilisation raisonnée des antibiotiques et qui affichent ainsi une faible prévalence d'infections bactériennes résistantes aux antibiotiques, il n'est guère besoin de nouveaux antibiotiques plus coûteux dont la supériorité thérapeutique par rapport aux composés existants n'a pas été démontrée. Étant donné que la résistance apparaît dès que l'on commence à utiliser un nouvel antibiotique, la désaffection de la Norvège pour les nouveaux antibiotiques concorde avec l'objectif d’en prolonger l'efficacité. Conséquence inattendue, le pays a signalé au secteur privé que ces nouveaux antibiotiques ne présentaient qu'un faible intérêt commercial mais qu'ils pourraient cependant être nécessaires pour traiter des maladies infectieuses rares ou nouvelles. Tous les pays souhaitent faire avancer la lutte contre les infections et promouvoir l'utilisation responsable des antibiotiques. Cependant, à mesure qu'ils progresseront, les bactéries résistantes aux antibiotiques devraient devenir moins fréquentes et la nécessité ainsi que l'intérêt commercial de nouveaux antibiotiques devraient par conséquent s'en trouver réduits. L'innovation en matière d'antibiotiques reste toutefois essentielle. Ce dilemme devra être résolu avec l'introduction de systèmes de récompenses alternatifs pour l'innovation dans le domaine des antibiotiques. Le consortium de recherche européen DRIVE-AB (*Driving re-investment in research and development and responsible antibiotic use*) a été chargé de trouver des solutions pour relever ce défi.

## Resumen

En este documento se detallan los procedimientos de autorización de comercialización y compensación para productos medicinales en Noruega, con especial referencia a nueve antibióticos nuevos que recibieron la autorización de comercialización entre 2005 y 2015. La descripción ilustra que, en lugares como Noruega, que cuentan con políticas eficaces de administración de antibióticos y una prevalencia baja relacionada de infecciones bacterianas resistentes a los antibióticos, hay poca necesidad de antibióticos nuevos más caros cuya superioridad terapéutica respecto a los componentes existentes no ha sido demostrada. Dado que la resistencia comienza a surgir tan pronto como se utiliza un antibiótico, la práctica de Noruega de dejar nuevos antibióticos de lado es coherente con el objetivo de prolongar la eficacia de los nuevos antibióticos. Una consecuencia inesperada es que el país ha explicado al sector privado que los antibióticos nuevos apenas tienen valor comercial, los cuales, sin embargo, pueden ser necesarios para tratar infecciones raras o nuevas. Todos los países tratan de mejorar su control de infecciones y fomentar un uso responsable de los antibióticos. No obstante, conforme se va progresando, las bacterias resistentes a los antibióticos deberían ser cada vez menos comunes y, por tanto, es probable que la necesidad y el valor comercial de los antibióticos nuevos se vean reducidos. Sin embargo, la innovación en el campo de los antibióticos sigue siendo fundamental. Este dilema deberá resolverse mediante la introducción de sistemas de recompensa alternativos por innovar con antibióticos. El consorcio de investigación europeo DRIVE-AB, que fomenta la reinversión en investigación y desarrollo y en el uso responsable de los antibióticos, deberá identificar formas de lograr este objetivo.

## ملخص

نتناول بالشرح التفصيلي في هذه الوثيقة إجراءات استرداد التكاليف والحصول على تصريح بتسويق المنتجات الدوائية في النرويج، مع الإشارة على وجه التحديد إلى تسعة أنواع من المضادات الحيوية الجديدة التي تم الحصول على تصريح بتسويقها في الفترة بين عامي 2005 و2015. ويبين هذا الشرح أنه في بعض الأماكن مثل النرويج – حيث تسري سياسات الإشراف على المضادات الحيوية وينخفض ما يرتبط بتلك الأدوية من انتشار للعدوى البكتيرية المقاومة للمضادات الحيوية – تقل الحاجة إلى مضادات حيوية جديدة أغلى سعرًا ولم يظهر دليل على تفوق أثرها عن المركبات الدوائية المتواجدة بالأسواق.

ونظرًا لبدء ظهور المقاومة بمجرد استخدام المضاد الحيوي، جاءت الممارسات التي اتبعتها النرويج – والتي تتمثل في التخلي عن المضادات الحيوية الجديدة – متسقةً مع الهدف الرامي إلى إطالة فترة فعالية المضادات الحيوية الجديدة. وأدى ذلك إلى نتيجة غير مقصودة حيث أوضحت النرويج للقطاع الخاص انخفاض القيمة التجارية للمضادات الحيوية الجديدة، والتي قد تظل الحاجة إليها قائمة رغم ذلك لعلاج أنواع العدوى الناشئة أو النادرة. وتسعى كل دولة إلى تحسين مكافحة العدوى وتعزيز الاستخدام المسؤول للمضادات الحيوية، غير أنه، مع المضي قدمًا في سبيل تحقيق ذلك، من المفترض أن يقل شيوع البكتريا المقاومة للمضادات الحيوية، وبالتالي، تظهر احتمالية أن تقل الحاجة إلى المضادات الحيوية الجديدة وأن تنخفض قيمتها التجارية. وبالرغم من ذلك، يظل ابتكار المضادات الحيوية يحتل مكانةً رئيسيةً. ويستلزم الأمر حل هذه المشكلة من خلال توفير نظم بديلة لمنح المكافآت فيما يتعلق بابتكار المضادات الحيوية. وقد تم تكليف اتحاد البحوث في أوروبا DRIVE-AB (القائم على توجيه أنشطة إعادة الاستثمار في مجال البحث والتطوير والاستخدام المسؤول للمضادات الحيوية) بمهمة تحديد سبل مواجهة ذلك التحدي.

## 摘要

本文中，我们会特别参考 2005 至 2015 年间获得上市许可的九种新型抗生素，详细地描述挪威医疗产品的上市许可及报销流程。该描述证明，像挪威这样拥有有效抗生素管理政策且耐抗生素细菌感染低相关发病率的地方，几乎不需要对现有化合物治疗优势尚未得到证明的更新、更昂贵的抗生素。 自抗生素使用之初耐药性便开始出现，挪威将新型抗生素束之高阁的做法符合延长新型抗生素效果的目标。 一个意料之外的后果是该国曾向私营部门发出这样的信号：新型抗生素毫无商业价值，但仍可能需要其治疗罕见或新型感染疾病。 每个国家都致力于改善感染控制并促进抗生素的负责任使用。 然而，随着不断进展，耐抗生素细菌感染率应有所减少，并且对新型抗生素的需求及其商业价值也可能会减少。 但是，抗生素的创新仍然至关重要。 这种困境必须通过引进替代性抗生素创新奖励机制进行解决。 位于欧洲的推动对抗生素研发及负责任使用再投资 (DRIVE-AB) 研究联合会已承担起寻找应对该挑战的方法的使命。

## Резюме

Здесь мы подробно описываем процедуры получения регистрационного удостоверения и финансового возмещения для лекарственных средств в Норвегии на примере девяти новых антибиотиков, которые получили регистрационное удостоверение между 2005 и 2015 гг. Описание демонстрирует, что в таких местах, как Норвегия, с эффективной стратегией рационального использования антибактериальных препаратов и со связанной с ней низкой распространенностью устойчивых к антибиотикам бактериальных инфекций практически нет необходимости в новых, более дорогих антибиотиках, терапевтическое превосходство которых не было продемонстрировано. Поскольку устойчивость начинает развиваться сразу при использовании антибиотика, практика Норвегии откладывать применение новых антибиотиков согласуется с целью продления эффективности новых антибиотиков. Непреднамеренным последствием является то, что страна дала понять малому бизнесу, что существует малая коммерческая ценность в новых антибиотиках, которые могут быть по-прежнему необходимы для лечения редких или новых инфекций. Каждая страна стремится усовершенствовать борьбу с инфекциями и стимулировать ответственное применение антибиотиков. Однако при достижении определенного прогресса устойчивые к антибиотикам бактерии должны стать менее распространенными и, как следствие, возможно, снизится потребность в новых антибиотиках и их коммерческая ценность. Тем не менее инновации антибиотиков продолжают оставаться значимыми. Эта дилемма должна быть решена за счет внедрения альтернативных систем вознаграждения за инновации антибиотиков. Исследовательскому консорциуму в Европе DRIVE-AB («Введение реинвестирования в научные исследования и разработки и ответственное использование антибиотиков») была поставлена задача определить пути решения этой проблемы.

## Introduction

Antibiotic-resistant bacteria that are harmful to humans are becoming more prevalent globally and are considered by many people to be one of the greatest threats facing our society today.^1^^,^^2^ Most worryingly, resistance to antibiotics of last resort has emerged, leaving the medical community with few or no tools to fight some pathogens.^3^ Moreover, antibiotic innovation by the private sector has dwindled to a low level in the past decade and currently only about five of the largest pharmaceutical companies are investing in antibiotic research and development.^4^

The DRIVE-AB (Driving re-investment in research and development and responsible antibiotic use) research consortium is financed through the New Drugs for Bad Bugs (ND4BB) programme by the European Union’s Innovative Medicines Initiative in partnership with members of the European Federation of Pharmaceutical Industries and Associations.^5^ The aim of the project is to develop, analyse and recommend policies that will stimulate innovation in antibiotic-related research and development while, at the same time, ensuring that any incentives will promote both the stewardship of antibiotics and equitable global access to them.

In this article, we examine the transition from marketing authorization to the use of novel antibiotics in one small country, Norway, which has five million inhabitants and one of the lowest per-capita utilization rates of antibiotics in the world.^6^^,^^7^ Although the Norwegian case is not generalizable to other countries, it provides a useful illustration of some unique characteristics of antibiotics markets and the resulting challenges faced by antibiotic innovators.

For this analysis, we focused on novel antibiotics that received marketing authorization in Norway between 2005 and 2015. An antibiotic was regarded as novel if no antibiotic with the same generic chemical name had previously received Norwegian marketing authorization. Several new formulations of older antibiotics, such as tobramycin, received marketing authorization between 2005 and 2015, but they were not considered novel. Information on novel antibiotics was obtained by searching the Norwegian Medicines Agency’s medicines database,^8^ the registry called Norwegian Prescription Database^9^ and the Norwegian drug wholesale statistics database^10^ and by consulting Norwegian community^11^ and hospital^12^ antibiotic guidelines. To understand the processes involved in, and the context of, marketing authorization and the utilization of novel antibiotics, we interviewed representatives, including doctors and pharmacists, of the Norwegian Medicines Agency, the Norwegian Antibiotic Centre for Primary Care and the Norwegian Competence Center for Antibiotic Use for Specialist Health Services.

## Marketing authorization and reimbursement

Norway is a member of the European Economic Area and participates in its common procedures for marketing authorization of medicinal products, which are coordinated by the European Medicines Agency (EMA). There are several ways of obtaining marketing authorization for pharmaceuticals in the European Economic Area. Typically, antibiotics are reviewed under the EMA’s so-called central procedure: an application for marketing authorization is sent to the EMA, which makes a binding decision for all countries in the European Economic Area based on the analyses of two national, regulatory agencies. Approval is valid for five years, after which the product must be reassessed. However, many older antibiotics received marketing authorization before the EMA’s central procedure had been established. As a result, there is some national variation across Europe in antibiotics that have been approved.

The Norwegian Medicines Agency is responsible for marketing authorization, pricing, reimbursement and other medicine-related services in Norway. Marketing authorization permits a medicinal product to be sold in community or hospital pharmacies but does not necessarily mean that its cost will be reimbursed by the Norwegian national health insurance scheme or that it will be used or purchased by health-care institutions. The national insurance scheme subsidizes the cost of prescription medicines prescribed through primary care to ensure that all critical medicines are accessible for Norwegian patients. Partial reimbursement of medication expenses in cases of severe or prolonged illness is included. Generally, the cost of antibiotics is not reimbursed because most infections are short-lived. In 2014, according to the Norwegian Prescription Database, only 13% of antibiotic prescriptions were reimbursed. However, some infections, such as methicillin-resistant *Staphylococcus aureus* (MRSA) infection, are regarded as so contagious and dangerous that the antibiotics used to treat them are always reimbursed.

**Fig. 1:**
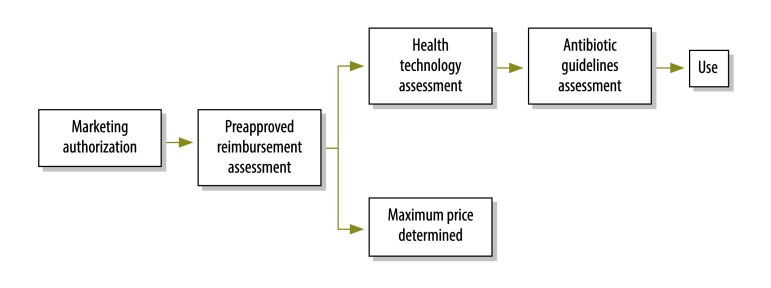
Flowchart from marketing authorization to antibiotic use, Norway

After a medicine has received marketing authorization in Norway, the manufacturer must apply separately to the Norwegian Medicines Agency for preapproval for reimbursement (Fig. 1). Subsequently, any primary care prescription for the medicine will be automatically eligible for reimbursement by the Norwegian national health insurance scheme. In its application for preapproval, the manufacturer must include documentation on the clinical effect of the drug, details of pharmacoeconomic analyses and price information. A health technology assessment is then performed. To be successful, the following four criteria must be met: (i) the medicine must treat a serious illness or reduce the risk of a serious illness relevant to Norwegian circumstances; (ii) the medicine must either be a long-term treatment or reduce the risk of long-term treatment (here, long-term is generally deemed 3 months or more); (iii) the medicine must have a well-documented, clinically relevant effect; and (iv) the medicine must be cost–effective according to appropriate health economic analyses.

Failure to meet any one of these criteria results in rejection of the application for reimbursement. As a result, if the medicine is prescribed by a community-based physician, the patient must bear the full cost out of pocket. However, the physician has the opportunity to apply for a patient-specific exception and, if approved, the medicine is covered by the national insurance scheme.

In parallel with the health technology assessment, the Norwegian Medicines Agency also determines a maximum price for the medicine. The agency uses an international reference pricing system, under which the manufacturer reports the price of the medicine for each indication in nine preselected countries: Austria, Belgium, Denmark, Finland, Germany, Ireland, the Netherlands, Sweden and the United Kingdom of Great Britain and Northern Ireland. The maximum price paid by the Norwegian national insurance scheme is the average of the three lowest prices applicable in these reference countries. Exchange rates are calculated using an average for the previous 6 months. If the application for reimbursement is approved, the manufacturer is permitted to sell its product in Norway at this maximum price or less.

Norwegian hospitals have formed a pooled procurement mechanism, by which medicines are purchased through national tenders. Having a maximum price is useful for judging the savings offered by these tenders and the mechanism has secured lower total costs for both generic and patented medicines. For patented medicines, savings are typically made on products that can be easily substituted. Nursing homes, which are included in community services, may competitively outsource the provision of their medicines. In all instances, no medicine may be sold in Norway at greater than its maximum price.

## National guidelines

Securing national insurance reimbursement does not necessarily mean that a medicine will be prescribed; that will depend on its inclusion in national guidelines and on physicians’ adherence to those guidelines. Norway has two sets of national guidelines for antibiotics – one for hospitals and one for community practice. Both are based on: (i) the evidence available, which is evaluated using the Grading of Recommendations Assessment, Development and Evaluation (GRADE) approach, where possible, to determine the quality of the evidence and the strength of the recommendations;^13^ (ii) antibiotic utilization data; (iii) epidemiological data; and (iv) national patterns of antibiotic resistance, including cross-resistance. Every year, the Norwegian Surveillance System for Antibiotic Resistance in Microbes and its veterinary counterpart report on national patterns of antibiotic resistance in humans and animals.^14^ Price is not taken into consideration in establishing community antibiotic guidelines. However, antibiotics used in the community tend to be older, generic drugs and are, therefore, inexpensive.

Community antibiotic guidelines are updated regularly by the Norwegian Antibiotic Centre for Primary Care. Although physicians are not legally required to follow the guidelines, they are expected to document their reasons for any antibiotic prescribing that differs substantially from the guidelines. Some deviation is acceptable and there are regional differences in prescribing, which suggests that the guidelines are either not adhered to fully or interpreted differently in different places.^14^ Substantial deviations in prescribing from guideline recommendations can have serious consequences: for example, one community physician lost his medical licence because of egregious deviations. Norway is considering introducing a peer-review system for monitoring the antibiotic prescribing habits of community physicians in which physicians would receive feedback on their prescribing habits from other physicians.

Hospital antibiotic guidelines are updated regularly by the National Competence Center for Antibiotic Use in Specialist Health Services. Although price is not taken into account during guideline development, it may influence the physician’s decision, especially when an antibiotic is substantially more expensive but has not demonstrated therapeutic superiority. Most new antibiotics are approved on the basis of clinical trials designed to establish noninferiority, which means that the new antibiotic is not inferior to a comparable existing antibiotic. It is difficult to demonstrate the superiority of an antibiotic because, in general, existing alternatives remain highly effective while antibiotic resistance is still quite uncommon. At present, older antibiotics continue to perform relatively well. In the absence of evidence of superiority, other factors, such as cost, potential side-effects and ease of administration, have a dominant influence on clinical decisions. The strategy of using patented, better-targeted antibiotics (for example, narrow-spectrum or pathogen-specific antibiotics) to slow the development of antibiotic resistance has, to date, not been a consideration in Norwegian hospital guidelines. The lack of certainty that resistance will emerge is too great to justify the high cost.

## Use of novel antibiotics

Nine novel antibiotics received marketing authorization in Norway between 2005 and 2015 (Table 1). All were approved via the EMA’s central procedure, except rifaximin, which was approved nationally. These antibiotics are rarely prescribed or used in Norway, although usage of a few, particularly rifaximin, was higher in 2015 than in 2014. Of the nine, six were preapproved for reimbursement from the national insurance scheme. The manufacturers of the remaining three (i.e. ceftolozane–tazobactam, fidaxomicin and retapamulin) have not applied to the Norwegian Medicines Agency for reimbursement. The cost of submitting an application for reimbursement for a novel medicine is 110 000 Norwegian kroner (approximately 12 750 United States dollars, US$) per indication. Because ceftolozane–tazobactam and fidaxomicin are primarily used in hospitals and because hospital budgets cover their cost, there is little reason for obtaining reimbursement authorization for primary care. Retapamulin is primarily prescribed in primary care but would be unlikely to meet the reimbursement criteria that it can be used to treat a serious illness or provide long-term treatment.

**Table 1:** Novel antibiotics awarded marketing authorization, Norway, 2005–2015

Generic chemical name	Brand name: formulation, package size	Type of antibiotic^a^	Reimbursement by Norwegian national insurance scheme	Year of marketing authorization^b^	Maximum price per package, US$^c^	Reimbursed amount per package, US$^c^	Included in community practice guidelines	Included in hospital guidelines	No. of packages sold in 2014^d^	No. of packages sold in 2015^d^
Ceftaroline fosamil	Zinforo: 600 mg, powder for infusion, 10 vials	Broad-spectrum	Yes	2012	985	985	No	No	39	42
Ceftolozane–tazobactam	Zerbaxa: 1 g ceftolozane and 0.5 g tazobactam, powder for infusion, 10 vials	Broad-spectrum	No	2015	1336	0	No	No	N/A	0
Daptomycin	Cubicin: 350 mg or 500 mg, powder for infusion, 1 vial^e^	Narrow-spectrum	Yes	2006	178^f^	178	No	Yes	482	646
Fidaxomicin	Dificlir: 200 mg, 20 tablets	Pathogen-specific	No	2011	1952	0	No	Yes	49	63
Levofloxacin^g^	Levofloxacin B.Braun: 5 mg/mL, solution for infusion, 20 × 100 mL vials	Broad-spectrum	Yes	2013	180	180	No	Yes	49	0
Retapamulin	Altargo: 1% ointment 5 g	Narrow-spectrum	No	2007	18	0	Yes	Yes	1708	2107
Rifaximin	Xifaxan: 550 mg, 56 tablets	Broad-spectrum	Yes	2013	390	390	No	No	338	896
Tedizolid	Sivextro: 200 mg, powder for infusion, 6 vials (or 6 200-mg tablets)	Narrow-spectrum	Yes	2015	1778^h^	1778	No	No	N/A	0
Tigecycline	Tygacil: 50 mg, powder for infusion, 10 vials	Broad-spectrum	Yes	2006	614	614	No	Yes	131	74

N/A: not applicable; US$: United States dollar.

^a^ A narrow-spectrum antibiotic is effective against only either Gram-negative or Gram-positive bacteria, whereas a broad-spectrum antibiotic is effective against both.

^b^ The year in which the antibiotic became available on the Norwegian market may have been later.

^c^ Prices have been converted from Norwegian kroner at a rate of 8 kroner to 1 United States dollar.

^d^ Data from the Norwegian drug wholesale statistics database.^10^

^e^ The 350-mg vial was withdrawn from the market in 2010.

^f^ Price is for the 500-mg vial.

^g^ Although levofloxacin received marketing authorization in the United States in 1996, it did not gain Norwegian marketing authorization until 2013. Some versions of levofloxacin without marketing authorization were sold in Norway in 2014 and 2015 – they are not included in the table.

^h^ Same price for both powder and tablets.

Note: Doripenem received marketing authorization in 2009 but was withdrawn from the market in 2014.

Of the nine antibiotics, three are considered narrow-spectrum antibiotics (i.e. daptomycin, retapamulin and tedizolid) and one is considered pathogen-specific (i.e. fidaxomicin against *Clostridium difficile*). In general, using a narrow-spectrum instead of a broad-spectrum antibiotic should reduce the likelihood of antibiotic-resistant bacteria developing, given that bacteria outside the spectrum of organisms covered would not be under selective pressure. Here, the use of these four antibiotics is examined in detail to give some insight into Norwegian physicians’ decision-making processes.

The pathogen-specific antibiotic fidaxomicin (maximum price in Norway: US$ 1952 per package) is normally used only in hospitals and nursing homes. In the guidelines it is listed as an alternative to vancomycin (maximum price: US$ 146 per package) for patients with recurrent *C. difficile* infections. Although vancomycin is a relatively narrow-spectrum antibiotic, it acts on a much broader range of organisms than fidaxomicin: it will kill several Gram-positive bacteria and, thereby, have an effect on normal microflora. However, given the price difference between the drugs and the low level of resistance to vancomycin (the level was assumed to be similar to that in Sweden^15^ since no Norwegian data were available), vancomycin was often prescribed in preference to fidaxomicin. In 2014, 1378 cases of *C. difficile* infection, including acute and recurrent infections, were reported to the Norwegian surveillance system for infectious diseases^16^ and 49 packages of fidaxomicin were sold.

Retapamulin (maximum price: US$ 18 per package) is a narrow-spectrum antibiotic for the topical treatment of bacterial skin infections. Interestingly, it was the most frequently prescribed of the nine novel antibiotics, although its use was still small, 2107 packages in 2015. The relatively high use was probably due to its inclusion in community guidelines as a topical treatment for impetigo, which is most likely to occur among preschool children. The alternative treatment is an antiseptic ointment containing dibrompropamindin.

Daptomycin (maximum price: US$ 178 per package) is listed in hospital guidelines as a treatment option for MRSA infections reported to the Norwegian Surveillance System for Infectious Diseases, typically as an alternative to vancomycin, as well as for vancomycin-resistant enterococci. In 2014, there were 833 cases of MRSA infection and 110 cases of infection with vancomycin-resistant enterococci. Health-care institutions dispensed 482 packages of daptomycin. Consequently, daptomycin probably has a substantial share of the small markets for these two indications.

Tedizolid (maximum price: US$ 1778 per package) is a narrow-spectrum antibiotic against acute, bacterial, skin and soft tissue infections by Gram-positive bacteria. It received marketing authorization in 2015, primarily on the basis of clinical trials showing it was noninferior to linezolid (maximum price: US$ 707 per package).^17^ By the end of 2015, tedizolid had not been used in Norway.

## Responsible use and innovation incentives

Human antibiotic use in Norway is among the lowest in the world:^7^ 15.1 defined daily doses per 1000 inhabitants per day in 2015.^14^ Yet the government, in its national strategy against antibiotic resistance for the period 2015 to 2020,^18^ adopted the goal that human antibiotic use in 2020 should be 30% lower than in 2012. Through strong infection control procedures and antibiotic stewardship measures, Norway has been successful in maintaining a low level of antibiotic resistance.^14^ Penicillin is still widely used and accounts for almost half of all antibiotics consumed – 41% of defined daily doses in 2015.^14^ As the prevalence of antibiotic-resistant bacteria is low in the country^14^ and given that existing antibiotics are still effective, there is little need to prescribe newer, more expensive drugs. Consequently, there is, so far, little or no market demand for newer antibiotics in Norway. Manufacturers may, therefore, rationally decide not to service the Norwegian market. Instead, they may give up almost entirely and focus their efforts on larger markets, such as those in the United States of America and European countries with higher levels of antibiotic resistance. For instance, between 2005 and 2015, the period we are considering in Norway, four additional novel antibiotics were approved in the United States: telavancin in 2009, dalbavancin and oritavancin in 2014 and ceftazidime–avibactam in 2015. None of these four has yet received marketing authorization in Norway. Two (dalbavancin and ceftazidime-avibactam) have been approved by the EMA in 2015 and 2016, respectively. The manufactures of the other two withdrew their EMA applications.

Nevertheless, antibiotic resistance is gradually becoming more common in Norway for most pathogens and, as a result, the pattern of antibiotic consumption has altered.^14^ For example, the use of narrow-spectrum penicillin decreased by 12% between 2000 and 2015.^14^ In addition, antibiotic-resistant bacteria can be imported from abroad. Swedish studies found that people travelling outside of northern Europe are often colonized by antibiotic-resistant bacteria.^19^^,^^20^ As antibacterial therapy must commence immediately after diagnosis, Norway must retain sufficient stocks of last-resort antibiotics to treat rare, multidrug-resistant infections. These stocks are expected to remain small. The result is that almost no revenues go from the Norwegian market to antibiotic innovators, which sends a signal to the private sector that there is little commercial value in novel antibiotics, at least in a market like Norway’s. However, Norway will probably still need novel antibiotics that can effectively treat rare, multidrug-resistant infections.

With current reimbursement models, it is difficult to provide an attractive return on investment in a country like Norway, where responsible use has diminished the size of the potential market for novel antibiotics to a negligible level. Today, however, probably only a handful of antibiotics markets resemble Norway’s. Yet, every country aspires to improve infection control and to promote responsible antibiotic use, thereby lowering the prevalence of antibiotic resistance. The need for novel, effective antibiotics will never disappear and there is the danger that, when a specific need arises in the future, it may be too late to develop novel drugs because medical innovation can be a lengthy process.

The DRIVE-AB consortium has been tasked with identifying ways of meeting the challenge of antibiotic innovation while at the same time promoting responsible use and equitable access. The proposed solutions are intended to reward innovation rather than boost sales and to increase both public and private investment in antibacterial research and development. The consortium’s final recommendations will be announced by October 2017.

## Conclusion

The Norwegian marketing authorization and reimbursement systems for medicinal products have been designed to ensure that Norwegians receive the medicines they need at the lowest possible price. Norwegian antibiotic guidelines have been devised to promote evidence-based prescribing in the context of the local pattern of antibiotic resistance. The spread of resistance in Norway has been slowed by strong stewardship policies and adherence to guidelines. This has enabled the country to leave newer antibiotics on the shelf to be used in an emergency for rare or emerging infections. The unintended consequence is that Norway has signalled to the private sector that there is little commercial value in novel antibiotics. The example of Norway demonstrates the need for alternative reward systems for antibiotic innovation.
